# Nurses’ professional competencies in urgency and emergency units: A mixed-methods study *

**DOI:** 10.1590/1518-8345.6554.3936

**Published:** 2023-05-15

**Authors:** Kemilys Marine Ferreira, Alexandre Pazetto Balsanelli, José Luís Guedes dos Santos

**Affiliations:** 1 Universidade Federal do Estado de São Paulo, Escola Paulista de Enfermagem, São Paulo, SP, Brasil.; 2 Universidade Federal de Santa Catarina, Departamento de Enfermagem, Florianópolis, SC, Brasil.

**Keywords:** Professional Competence, Emergency Nursing, Leadership, Nursing Administration Research, Emergency Medical Services, Nurse’s Role, Competencia Profesional, Enfermería de Urgencia, Liderazgo, Investigación en Administración de Enfermería, Servicios Médicos de Urgencia, Rol del Enfermero, Competência Profissional, Enfermagem em Emergência, Liderança, Pesquisa em Administração de Enfermagem, Serviços Médicos de Emergência, Papel do Profissional de Enfermagem

## Abstract

**Objective:**

to describe the professional competencies of nurses working in urgency and emergency services and to understand their perception of the essential competencies for professional performance and updating.

**Method:**

a sequential, mixed-methods and explanatory study, conducted with emergency nurses. To obtain the quantitative data, a questionnaire with 78 items was used, answered by 39 nurses and analyzed using descriptive statistics and non-parametric tests. In turn, the qualitative data were obtained through semi-structured interviews with 17 nurses, whose interpretation was based on inductive content thematic analysis. The data were combined by connection.

**Results:**

a high level of competence was obtained in the self-assessment of urgency and emergency nurses in Factor 2 - Relations at work and a lower level in Factor 6 - Professional excellence (p=0.036). The qualitative data corroborated positively with the “Relations at work” factor, identifying the association of knowledge and practical experience, with competencies beyond a scenario devoid of permanent education.

**Conclusion:**

despite the high levels of competence identified in emergency nurses, the strengthening of educational strategies favors professional development and recognition.

## Abstract (es)

**Objetivo:**

describir las competencias profesionales de los enfermeros que trabajan en urgencias y emergencias y comprender la percepción que tienen sobre las competencias esenciales para el desempeño y la actualización profesional.

**Método:**

estudio secuencial explicativo de métodos mixtos, realizado con enfermeros de urgencias y emergencias. Para obtener datos cuantitativos, se utilizó un cuestionario con 78 ítems, respondido por 39 enfermeros, que fueron analizados mediante estadística descriptiva y pruebas no paramétricas. Los datos cualitativos se obtuvieron a través de entrevistas semiestructuradas con 17 enfermeros, que fueron interpretados mediante el análisis temático de contenido inductivo. Los datos se combinaron por conexión.

**Resultados:**

se obtuvo un alto nivel de competencia en la autoevaluación de los enfermeros de urgencias y emergencias en el factor 2 - relaciones en el trabajo y un nivel más bajo en el factor 6 - excelencia profesional (p=0,036). Los datos cualitativos coincidieron positivamente con el factor relaciones en el trabajo, identificando la asociación entre conocimiento y experiencia práctica con las competencias, además de un escenario que carece de educación continua.

**Conclusión:**

a pesar de los altos niveles de competencia identificados en los enfermeros de urgencias y emergencias, el fortalecimiento de las estrategias educativas favorece el desarrollo y reconocimiento profesional.

## Abstract (pt)

**Objetivo:**

descrever as competências profissionais dos enfermeiros que atuam em urgência e emergência e compreender sua percepção a respeito das competências essenciais para a atuação e a atualização profissional.

**Método:**

estudo sequencial explanatório de métodos mistos, realizado com enfermeiros de urgência e emergência. Para a obtenção dos dados quantitativos, utilizou-se um questionário com 78 itens, respondidos por 39 enfermeiros, que foram analisados mediante estatística descritiva e testes não paramétricos. Já os dados qualitativos, foram obtidos por entrevista semiestruturada realizada com 17 enfermeiros, cuja interpretação deu-se pela análise temática de conteúdo indutiva. Os dados foram combinados por conexão.

**Resultados:**

obteve-se alto nível de competência na autoavaliação dos enfermeiros de urgência e emergência no fator 2 – relações no trabalho e menor nível no fator 6 - excelência profissional (p=0,036). Os dados qualitativos corroboraram de forma positiva com o fator relações no trabalho, identificando a associação do conhecimento e experiência prática com competências, além de um cenário desprovido de educação permanente.

**Conclusão:**

apesar de altos níveis de competência identificados nos enfermeiros de urgência e emergência, o fortalecimento de estratégias educacionais favorece o desenvolvimento e o reconhecimento profissional.

**Highlights:**

**(1)** Emergency nurses demonstrated a high level of professional competence.

**(2)** The absence of Permanent Education affected nurses’ professional competencies.

**(3)** Recognition occurs with knowledge, experience and interpersonal relationships.

**(4)** Permanent Education in the emergency sector can develop professional competencies.

## Introduction

The health demands of the population are oftentimes confronted in the urgency and emergency sector, where fast, dynamic and decisive care is sought ^( 1)^ . These situations require immediate care and, therefore, duly trained and qualified professionals capable of offering effective, systematic and competent care ^( 2)^ . In this context, nurses need to develop competencies to provide and promote safe, excellent and humanized Nursing care, guided by leadership and management of effective processes and material resources ^( 2)^ . 

The concept of professional competence involves knowledge, experiences and personal values, as well as people’s way of acting while performing their work ^( 3- 4)^ . In this sense, nurses guide their own care actions through a profile of competencies established in the National Curriculum Guidelines ( *Diretrizes Curriculares Nacionais*, DCN) for undergraduate Nursing courses ^( 5)^ . 

The competencies developed by nurses go through an educational process that requires continuous updating, given the innovations in the health area. In relation to professional improvement, a study showed that, in the face of emergency care, fragmented educational actions can generate professional dissatisfaction and reduce the level of competence ^( 6)^ . As it is a challenging issue in Nursing, in addition to reducing job satisfaction, the feeling of low competence can increase absenteeism and affect the quality of the care provided ^( 7)^ . 

Thus, it becomes necessary to map professional competences in Nursing, given that nurses are confronted every day with the competencies they already have and with the need to develop others, necessary for their practice, as they are interconnected to the health care results ^( 8)^ . However, identifying and measuring them is both a major challenge ^( 9)^ and an ongoing need. 

That said, this study may contribute to the teaching of the professional Nursing practice in emergency sectors, as the mapping of competencies favors a diagnostic analysis that identifies the weaknesses to be worked on through educational programs, with a view to benefiting health care.

The following was adopted as guiding question: Which are the professional competencies of nurses working in the urgency and emergency context? And what would be these nurses’ understanding regarding the competencies required for the professional practice? Thus, the objective of this study was to describe the professional competencies of nurses working in urgency and emergency sectors services and to understand their perception of the essential competencies for professional performance and updating.

## Method

### Study design

This is a mixed-methods study with an explanatory and sequential design. This approach is characterized by the greater weight attribution to the quantitative stage, whose data are collected and analyzed first. In this sequence, the qualitative stage is developed in order to deepen the initial quantitative data ^( 10)^ . In synthesis, this type of study introduces a qualitative element to explain the initial quantitative results ^( 11)^ . Data combination occurred through connection, where the results in a first analysis inform sampling to the other. In this case, the quantitative results informed the objectives of the qualitative sampling procedure ^( 12)^ . 

The quantitative stage was performed from a descriptive-analytical cross-sectional study, outlined according to Strengthening the Reporting of Observational Studies in Epidemiology (STROBE). The qualitative phase was characterized as an exploratory-descriptive research study, guided by the Consolidated Criteria for Reporting Qualitative Research (COREQ). In the mixed-method approach, the Mixed Methods Appraisal Tool (MMAT) was used as instrument for analyzing methodological rigor ^( 13)^ . 

For reliability of the qualitative data, the three authors discussed and validated the topics and subtopics from the participants’ testimonies, collected from semi-structured interviews. The choice to use a mixed methodology also contributed to reliability of the qualitative data, as it was possible to identify convergences with the quantitative results initially obtained and expand understanding of the phenomenon investigated.

### Data collection scenario

The study was carried out within the scope of the secondary and tertiary health care levels, including two 24-hour Emergency Care Units ( *Unidades de Pronto Atendimento*, UPA), a Mobile Emergency Care Service ( *Serviço de Atendimento Móvel de Urgência*, SAMU) and three General Hospitals, located in two municipalities from the South of the state of Minas Gerais, Brazil. 

### Population

The population of the quantitative stage involved 56 nurses who worked in the services selected for research linked to the Urgency and Emergency Network ( *Rede de Urgência e Emergência*, RUE). 

The quantitative data analysis, performed via the Mann-Whitney U test, identified that secondary care nurses had lower levels of competence. Based on this result, the participants of the qualitative stage were defined, as provided for in the mixed design adopted ^( 10)^ . Thus, the population of the qualitative stage of the study consisted of 24 nurses from the secondary care urgency and emergency units (UPA and SAMU) that participated in the first stage of the study. 

### Selection criteria

Nurses who had at least six months of experience in the urgency and emergency sectors were included in the quantitative stage of the study. The nurses that had participated in the first stage were included in the qualitative stage. Those who were absent during the data collection period due to vacation or extended leave were excluded from both stages.

### Definition of the sample

The type of sample selected for the research was non-probabilistic, for convenience and resulted in 39 participants from the 56 nurses in the quantitative phase. The losses were due to layoff of professionals from the urgency and emergency services during data collection and to the participants not returning the instrument within 20 days. In the qualitative stage, of the 24 nurses, only 17 participants comprised the sample, which was intentional. From participant N17, the data saturation scope was perceived, thus interrupting the scheduling of new interviews.

### Instruments used to collect the information

The Competence Scale of Actions of Nurses in Emergencies ( *Escala de Competências das Ações dos Enfermeiros em Emergências*, ECAEE) ^( 14)^ was used in the quantitative study, a validated national instrument consisting of five parts: Part A - Sociodemographic characteristics; Part B - Professional training; Part C - Parameters of emergency nurses’ level of competence. These marked the levels of competence of nurses working in emergencies. The score varied from 1 to 5 points from “Not at all competent” to “Extremely competent” ^( 9)^ ; they were frameworks, similar to a Likert scale, for the participants to answer parts D and E of the instrument; Part D - Assessment of the competence level presented by nurses in fictitious situations, where the participants should indicate such level from 1 to 5 (1 - Not at all competent, 2 - Little competent, 3 - Competent, 4 - Very much competent and 5 - Extremely competent), that best portrayed the reality of the nurses’ work described, according to their professional perception. Finally, Part E, which consisted of 78 items that represented the actions for the practice of nurses working in emergencies, raising a self-assessment of the in-service practice. 

ECAEE is supported by the professional competence matrix of nurses working in emergencies, consisting of 8 basic competencies and 32 associated competencies ^( 14)^ . 

The content expressed in Part E of the instrument was validated for seven competence factors and the items that comprise them, namely: Factor 1 - Professional practice (33 items); Factor 2 - Relationships at work (19 items); Factor 3 - Positive challenge (10 items); Factor 4 - Targeted action (7 items); Factor 5 - Constructive behavior (2 items); Factor 6 - Professional excellence (4 items) and Factor 7 - Adaptation to change (3 items) ^( 15)^ . However, these factors are not explained in the printed tool, in Part E only the 78 items are listed. Calculation of the level of competence is made based on the values of the scores resulting from the mathematical operations of addition of each of the seven factors ^( 9)^ . 

In the qualitative study, a script was used for the semi-structured interview, prepared by the authors and which included sample characterization data and dissertation questions that addressed the participants’ perception of the competencies required to achieve professional recognition, strategies for updating knowledge in the emergency area and the permanent education scenario in the work unit. The script was previously submitted to a pilot test with a nurse from the UPA who had not participated in the first stage because she was on maternity leave. The participant was invited, signed the Free and Informed Consent Form, was explained the first stage of the research, as well as she was informed about the reasons for the need to carry out the qualitative stage. The test was not considered in the analyses.

### Data collection

The quantitative stage took place between July and October 2020 from self-completion of the ECAEE instrument ^( 14)^ . The participants were free to choose to answer the instrument at the time of delivery, during work or to take it home and return it completed within 20 days. 

The qualitative stage took place from June to August 2021, based on semi-structured individual interviews previously scheduled with the participants and recorded through an iPad voice recorder (chip-free, without access to social networks or messaging apps). They were carried out by one of the authors during the working hours and within the emergency sector, in a private environment chosen by the participants themselves. They lasted a mean of 10 minutes.

The occurrence of bias in the quantitative stage may have been due to the length of the questionnaire; in turn, in the qualitative stage, it may have been due to the time of the interviews and the audible alarms of the devices in the emergency services. When there was an interruption due to alarms or at the request of the team to the participant, the recording was paused and restarted later. In both stages, the data were collected during the COVID-19 pandemic, a scenario in which the professionals were fully involved in coping with this health emergency. Despite this reality, the nurses were continuously encouraged to contribute to the study, understanding that the benefits for their professional future would outdo the risks.

### Data treatment and analysis

The quantitative data were entered into spreadsheets; there was no double-typing due to the number of participants (n=39) in the quantitative stage. However, the entire transcription of the data to the database was carried out with utmost care so that all the pieces of information were in fact those collected. Subsequently, descriptive and inferential statistical analysis was performed using the Statistical Package for the Social Sciences ^®’^(SPSS ^®^) software, version 25.0. 

In the data analysis, the quantitative variables (sex, workplace and age) were presented as relative frequencies. For the analysis of the score obtained in the competence factors of the self-assessment, descriptive statistics with absolute and relative frequencies was used. The Mann-Whitney U test was employed to compare the groups of participants in secondary (UPA and SAMU) and tertiary (General Hospitals) care regarding the score classification in the factors. A non-parametric test was chosen because it allows comparing asymmetric data from the same population and verifying similarities between them.

The statistical significance value adopted was 5% (p≤0.05). The absolute and relative frequency tables presented about the factors, describing the degree of competence and actions of nurses working in emergencies, favor readers to quickly identify the decline in items 4 and 31 from Factor 6 - Professional excellence.

The calculation described in Part E of the instrument refers to an operation of adding the degree of competence marked in each item by the participant; adding the degrees to obtain the result corresponding to the level of competence. This calculation leads to the same result of the analysis applied to the study.

In the qualitative stage, the interviews were initially transcribed *verbatim* and later adapted with spelling correction. Tables with topics were organized according to the issues being evaluated based on the Thematic Content Analysis, with an inductive approach ^( 15- 16)^ . The manually performed coding evidenced thematic categories that were discussed and validated by all three authors, ensuring reliability of the study. 

The combination between quantitative and qualitative results was carried out through the elaboration of joint-displays aiming at the connection between the data of the two approaches ^( 12)^ . This strategy enabled the elaboration of meta-data, results of the combination of quantitative and qualitative data. 

### Ethical aspects

This study was approved by the Ethics Committee of the Federal University of the State of São Paulo ( *Universidade Federal de São Paulo*) under opinion No. 4,099,526. All ethical principles set forth in Resolutions 466/2012 and 510/2016 of the National Health Council. During the first research stage, the participants were presented to the Free and Informed Consent Form as well as the study objectives and, after their consent, two copies were signed. There was a specific field in which the participants indicated their consent to participate in the second stage, the qualitative one. The participants’ identity was hidden by the letter N (Nurse), followed by the number corresponding to the interview in the data collection order. 

## Results

The quantitative study was carried out with 39 urgency and emergency nurses, with predominance of the female gender (84.6%), working in secondary (61.5%) and tertiary health care (38.4%). Of these, 48.7% worked in UPA, 12.8% in the SAMU and 38.4% in General Hospitals, with a working time in the area of 4 to 7 years (45.9%) and a graduate degree in the emergency area (76.4%). The age group varied from 20 to 63 years old, with a mean of 37.9 and a median of 35.

In relation to the self-assessment, the nurses presented Level 4/Very much competent according to the score of the ECAEE ^( 10)^ , in the following competence factors: 1 - Professional practice, 2 - Relationships at work, 3 - Positive challenge, 4 - Targeted action, 5 - Constructive behavior and 7 - Adaptation to change. However, in the self-assessment of Factor 6 - Professional excellence, especially in items 4 and 31, a decline was noted, predominantly Level 3/Competent, as shown in Table 1. 

**Table 1 -:** Actions of Nurses in Emergency in Factor 6 - Professional excellence, among the study participants (n=39) in two municipalities from the South of the state of Minas Gerais, Brazil, 2020

N*	**Representative items of nurses’ actions in emergencies**	**Competence Level (n=39)**				
		**1** ***n**_i_* ^†^ ***f**_i_* **(%)** ^‡^	**2** ***n**_i_* ^†^ ***f**_i_* **(%)** ^‡^	**3** ***n**_i_* ^†^ ***f**_i_* **(%)** ^‡^	**4** ***n**_i_* ^†^ ***f**_i_* **(%)** ^‡^	**5** ***n**_i_* ^†^ ***f**_i_* **(%)** ^‡^
**4**	Constantly updates knowledge in emergencies.	0	8 (20.5)	13 (33.3)	12 (30.7)	6 (15.3)
**29**	Makes a Nursing diagnosis for the client according to the theoretical framework adopted in the institution.	0	3 (7.6)	12 (30.7)	17 (43.5)	7 (17.9)
**31** ^§^	Participates in realistic simulation in emergencies periodically.	2 (5,2)	11 (28,9)	18 (47,3)	5 (13,1)	2 (5,2)
**55**	Uses other opportunities for their professional development.	0	0	16 (41.0)	16 (41.0)	7 (17.9)

^*^N = Number corresponding to the item representing the actions of nurses in emergency; ^†^ *n _i_* = Absolute frequency of the participants’ answers; ^‡^ *f _i_* (%) = Relative frequency (in percentage) of the participants’ answers; ^§^1 loss – the participant did not return the answers

Differently, Factor 2 - Relationships at work, stood out positively in the analysis of the self-assessments among the other factors, in which all items representing nurses’ actions in emergencies presented higher frequencies of Level 4/Very much competent, according to Table 2. 

**Table 2 -:** Actions of nurses in Emergency in Factor 2 - Relationships at work, among the study participants (n=39) in two municipalities from the South of the state of Minas Gerais, Brazil, 2020

N*	**Representative items of nurses’ actions in emergencie**s	**Competence Level (n=39)**				
		**1** ***n**_i_* ^†^ ***f**_i_* **(%)** ^‡^	**2** ***n**_i_* ^†^ ***f**_i_* **(%)** ^‡^	**3** ***n**_i_* ^†^ ***f**_i_* **(%)** ^‡^	**4** ***n**_i_* ^†^ ***f**_i_* **(%)** ^‡^	**5** ***n**_i_* ^†^ ***f**_i_* **(%)** ^‡^
**2**	Maintains emotional control when deploying solutions to problems.	0	1 (2.56)	11 (28.21)	19 (48.72)	8 (20.51)
**5** ^§^	Achieves agreements at work by resorting to dialog.	0	1 (2,63)	9 (23,68)	19 (50,00)	9 (23,68)
**14**	Acts without prejudging people.	0	2 (5.13)	11 (28.21)	17 (43.59)	9 (23.08)
**18**	Conveys a message without distorting its content with the means available to do so.	0	0	10 (25.64)	20 (51.28)	9 (23.08)
**19**	Makes people feel like they are part of a group.	0	0	5 (12.82)	20 (51.28)	14 (35.90)
**22**	Acts within the limits of the ethics required by the globalized world when communicating.	0	0	10 (25.64)	17 (43.59)	12 (30.77)
**23**	Has transparent, honest and responsible behaviors in relationships with people.	0	0	5 (12.82)	19 (48.72)	15 (38.46)
**29**	Listens to people without prejudging their ideas and opinions.	0	2 (5.13)	10 (25.64)	18 (46.15)	9 (23.08)
**43**	Maintains a healthy professional relationship with people.	0	4 (10.26)	15 (38.46)	16 (41.03)	4 (10.26)
**48**	Avoids conflicting behaviors with people.	1 (2.56)	2 (5.13)	10 (25.64)	16 (41.03)	10 (25.64)
**49**	Clarifies other people’s doubts according to needs.	0	0	8 (20.51)	20 (51.28)	11 (28.21)
**55**	Accepts people as they are.	0	1 (2.56)	11 (28.21)	16 (41.03)	11 (28.21)
**63**	Guarantees expression of each person’s will.	0	4 (10.26)	12 (30.77)	18 (46.15)	5 (12.82)
**65**	Seeks to establish harmonious contact with the other.	0	1 (2.56)	6 (15.38)	21 (53.85)	11 (28.21)
**66**	Identifies the action freedom limit they have.	0	2 (5.13)	7 (17.95)	22 (56.41)	8 (20.51)
**69**	Listens to people with a clear interest in fulfilling their requests.	0	1 (2.56)	7 (17.95)	17 (43.59)	14 (35.90)
**76**	Proposes adjustments without generating conflicts.	0	0	11 (28.21)	20 (51.28)	8 (20.51)
**78**	Maintains a good relationship when managing emotions at work.	1 (2.56)	0	14 (35.90)	16 (41.03)	8 (20.51)

^*^N = Number corresponding to the item representing the actions of nurses in emergency; ^†^ *n _i_* = Absolute frequency of the participants’ answers; ^‡^ *f _i_* (%) = Relative frequency (in percentage) of the participants’ answers; ^§^1 loss – the participant did not return the answers

Table 3 presents the distribution of the participants in relation to the classification of the score obtained in each factor in the self-assessment according to the type of performance (secondary or tertiary care). 

**Table 3 -:** Comparison of the participants (n=39) regarding the types of performance (secondary and tertiary care) in relation to the classification of the scores obtained in the competence factors, in two municipalities from the South of the state of Minas Gerais, Brazil, 2020

Factor*	**Classification**	**Type of performance**						**p** ^§^	**E.S.** ^||^
		**Secondary**		**Tertiary**		**Total**			
		***n**_i_* ^†^	***f**_i_* **(%)** ^‡^	***n**_i_* ^†^	***f**_i_* **(%)** ^‡^	***n**_i_* ^†^	***f**_i_* **(%)** ^‡^		
**1**	Not at all competent	0	0.00	0	0.00	0	0.00	0.887	0.031
	Little competent	0	0.00	0	0.00	0	0.00		
	Competent	1	4.17	1	6.67	2	5.13		
	Very much competent	13	54.17	7	46.67	20	51.28		
	Extremely competent	10	41.67	7	46.67	17	43.59		
**2**	Not at all competent	0	0.00	0	0.00	0	0.00	0.173	0.230
	Little competent	0	0.00	0	0.00	0	0.00		
	Competent	1	4.17	0	0.00	1	2.56		
	Very much competent	14	58.33	6	40.00	20	51.28		
	Extremely competent	9	37.50	9	60.00	18	46.15		
**3**	Not at all competent	0	0.00	0	0.00	0	0.00	0.216	0.187
	Little competent	0	0.00	0	0.00	0	0.00		
	Competent	3	12.50	3	20.00	6	15.38		
	Very much competent	17	70.83	5	33.33	22	56.41		
	Extremely competent	4	16.67	7	46.67	11	28.21		
**4**	Not at all competent	0	0.00	0	0.00	0	0.00	0.532	0.095
	Little competent	0	0.00	0	0.00	0	0.00		
	Competent	2	8.33	2	13.33	4	10.26		
	Very much competent	16	66.67	7	46.67	23	58.97		
	Extremely competent	6	25.00	6	40.00	12	30.77		
**5**	Not at all competent	0	0.00	0	0.00	0	0.00	0.168	0.227
	Little competent	0	0.00	1	6.67	1	2.56		
	Competent	4	16.67	1	6.67	5	12.82		
	Very much competent	14	58.33	5	33.33	19	48.72		
	Extremely competent	6	25.00	8	53.33	14	35.90		
**6**	Not at all competent	0	0.00	0	0.00	0	0.00	**0.036** ^§^	0.340
	Little competent	1	4.17	0	0.00	1	2.56		
	Competent	12	50.00	3	20.00	15	38.46		
	Very much competent	9	37.50	9	60.00	18	46.15		
	Extremely competent	2	8.33	3	20.00	5	12.82		
**7**	Not at all competent	0	0.00	0	0.00	0	0.00	0.973	0.010
	Little competent	0	0.00	0	0.00	0	0.00		
	Competent	6	25.00	4	26.67	10	25.64		
	Very much competent	14	58.33	8	53.33	22	56.41		
	Extremely competent	4	16.67	3	20.00	7	17.95		
**Total**	Not at all competent	0	0.00	0	0.00	0	0.00	0.295	0.166
	Little competent	0	0.00	0	0.00	0	0.00		
	Competent	0	0.00	1	6.67	1	2.56		
	Very much competent	18	75.00	7	46.67	25	64.10		
	Extremely competent	6	25.00	7	46.67	13	33.33		

^*^Factor 1 - Professional practice; Factor 2 - Relationships at work; Factor 3 - Positive challenge; Factor 4 - Targeted action; Factor 5 - Constructive behavior; Factor 6 - Professional excellence; Factor 7 - Adaptation to change; ^†^ *n _i_* = Absolute frequency of the participants’ answers; ^‡^ *f _i_* (%) = Relative frequency (in percentage) of the participants’ answers; ^§^p = p- value at the 5% level (p≤0.05); ^||^E.S. = Effect Size

The results in Table 3 show that there was a difference between the groups of participants in relation to the score classification only for Factor 6 (p=0.036), with individuals who worked in tertiary care presenting better score classifications when compared to those who worked in secondary care. No differences were observed between the groups for the other factors. 

From this finding, it was sought to understand the nurses’ perception that was related to the lower self-assessment score in this factor. Thus, the qualitative stage was performed with a sample comprised by 17 nurses from the secondary health care units (UPAs and SAMU). These participants were mostly female (82.3%), aged from 40 to 50 years old (47.0%) and with a working time from 5 to 7 years (64.7%).

The data content thematic analysis enabled the construction of two thematic categories. In the first category, “Professional recognition of nurses by the labor market”, it was noticed that, to achieve professional recognition, it was necessary to constantly seek knowledge, associated with the practical experience acquired at work and the ability to develop interpersonal relationships, as illustrated by the testimonies below.

*So I believe that it’s enough, but we can’t stay still, we have to always seek new knowledge.* (N2)

*[...] always updating and aiming at an improvement in all these factors, to qualify and add more quality to our service.* (N13)

*[...] I would actually say two things: knowledge and practice! If you don’t have knowledge combined with practice, it’s difficult. Only knowledge becomes difficult, if you don’t have practice, knowledge alone ties you up, practice makes you put that knowledge to work in favor of others, doesn’t it?* (N17)

*I think that, to be a complete professional, we need to have within these approaches, as there’s no way you can have a technically qualified professional if they don’t have a good relationship, right?* (N7)

The second category, “Deficient permanent education scenario in urgency and emergency services”, presented a deficient Permanent Education context. In most cases, 15 (88.0%) of the testimonies reported absence of Permanent Education in their work unit.

Regarding the main professional updating method, the participants reported attending private courses in the online modality. Based on the perception of deficits, the participants suggested proposals to improve the scenario, such as the following: organization of a Permanent Education team for training in health services in a systematic way, in addition to investments in human and financial resources for structuring the SAMU Center for Education in Urgencies ( *Núcleo de Educação em Urgência*, NEU), thus enabling practical training with realistic simulation, as shown in the testimonies below:

*There’s no permanent education scenario, until today there is not, we do it on the Internet, it’s for* *us ...*(N1)

*The Unified Health System (*Sistema Único de Saúde *, SUS), differently from a private network, is very outdated in terms of training, I think it should have more training, they should invest more in the professional. Because I see that in the private network they focus more on training, there are lectures, there’s continuing education... The people of the public system, the system is still outdated. So if we don’t look for an update on our own, I think we end up wanting it, you know?* (N2)

*At the moment we don’t have a permanent education scenario, we’re trying to develop it, but in these 15 years of the SAMU it’s always been very flawed.* (N6)

*Those who have training today are on their own merit [...] I try to update with Internet courses and by indication from peers. But very fast things, with small hour loads.* (N7)

*I think that there should be a team to do this permanent education of the staff. To have a commission, a team for this, that would be directed to take this education to the staff.* (N9)

*We’ve created our center for education in urgency (NEU), it was created already two or three years ago. We’re working on elaboration of the protocols, we already have a room, we already have everything prepared for this. However, there’s still a lot of material, a lot of commitment from the city to buy all the equipment [...] which would be more important than the investment in permanent education, purchase of general material from audiovisual to simulators, everything!* (N16)

For data integration, two joint-displays were prepared, as presented below. The first one shows the results of the self-assessment of the secondary care participants in Factor 6 - Professional excellence related to the score obtained in the thematic categories Figure 1. The second one indicates the meta-data derived from the testimonies of the secondary care participants related to the score obtained with the self-assessment of Factor 2 - Relationships at work, in the secondary and tertiary care group Figure 2. 

**Figure 1 -:** Self-assessment of the secondary care participants (n=17) in Factor 6 - Professional excellence, related to the scores obtained in the Thematic Categories and Meta-inferences. Municipalities from the South of the state of Minas Gerais, Brazil, 2020 and 2021

**ITENS**	QUAN* RESULTS	**QUAL** ^†^ **RESULTS**	**META-INFERENCES**
33. Uses other opportunities for their professional development	**50.0% of the nurses obtained Level of Competence 3 (Competent)** *versus* **220.0% of tertiary care** **p** ^‡^ **= 0,036**	Category 1 - Professional recognition of nurses by the labor market	There is an association in the participants’ discourse that theoretical knowledge and practical experience are directly related to competence. The interpersonal relationship is highlighted as a “key” to professional recognition. There is a precarious permanent education scenario in the emergency units, which leads professionals to seek individual training according to the work need. The participants seek emergency training through private means, mainly in digital platforms and online courses. There is no practical training or realistic simulation in the emergency services surveyed. Investment in human resources aimed at organizing a permanent education team, in addition to financial investment to purchase equipment to carry out practical training, which are strategies proposed by the participants to improve the current deficient scenario.
3. Constantly updates knowledge in emergencies. 4. Participates in realistic simulation in emergencies periodically		Category 2 - Permanent education deficient scenario of urgency and emergency services	

^*^QUAN = Quantitative; ^†^QUAL = Qualitative; ^‡^p = p-value

**Figure 2 -:** Self-assessment of the secondary care participants (n=17) in Factor 2 - Relationships at work, related to the score obtained (4 - Very much competent), the participants’ testimonies and the Meta-inferences. Municipalities from the South of the state of Minas Gerais, Brazil, 2020 and 2021

**ITENS**	QUAN* RESULTS	**QUAL** ^†^ **RESULTS**	**META-INFERENCES**
2. Maintains emotional control when deploying solutions to problems.	***f*** ^‡^ **=19 (48,72%)**	Secondary care nurses interviewed (n ^§^=17) Y *ou have to be agile, calm, you can’t get too anxious, because in the emergency sector you have to know how to control anxiety, nervousness, stress.* (N9) [...] *if we’re unable to have a good interpersonal or multiprofessional relationship, this is more difficult.… Because everyone learns to puncture a vein. It may take a little longer, or a little less, but everyone learns. But now, we make one person know how to deal with the other, then we already deal with other issues that are more personal, so I believe that what is more fundamental for a professional is the issue of good relationships, peers, team and patient alike, which is difficult.* (N7)	The testimonies corroborate the positive self-assessment of the participants in Factor 2 - Relationships at work. Communication skills are directly related to interpersonal relationships at work. These relationships at work should be developed respecting the different opinions and practicing emotional control in the face of adversity.
5. Achieves agreements at work by resorting to dialog.	***f*** ^‡^ **=19 (50,00%)**		
18. Conveys a message without distorting its content with the means available to do so.	***f*** ^‡^ **=20 (51,28%)**		
19. Makes people feel like they are part of a group.	***f*** ^‡^ **=20 (51,28%)**		
65. Seeks to establish harmonious contact with the other.	***f*** ^‡^ **=21 (53,85%)**		
66. Identifies the action freedom limit they have.	***f*** ^‡^= **22 (56,41%)**	*You need to have a connection among the team members [...] and we need to have the same words, speak the same language, right?! So I think a good team, a good service, it has to be quite cohesive.* (N8) *Nursing is a difficult profession from another point of view, because we’re already trained knowing that, unlike most of society, we won’t have Saturdays, Sundays or free holidays, we’d have a lot of work for some 30-35 years with Saturdays, Sundays and working holidays. And seek to create a healthy environment within the workplace.* (N11)	For the participants, in addition to providing a less hostile and more pleasant work environment for all, interpersonal relationships reflect greatly on the satisfaction of the Nursing leader and the team, resulting in a high quality of the care provided to the patients.
76. Proposes adjustments without generating conflicts.	***f*** ^‡^= **20 (51,28%)**		

^*^QUAN = Quantitative; ^†^QUAL = Qualitative; ^‡^ *f* = Frequency; ^§^ *n* = Number of participants

## Discussion

The results obtained indicate predominance of the female gender, strengthening the concept that Nursing is a profession mostly developed by women, representing 89% of the profession’s workforce, with variations between regions of the world ^( 17)^ and mostly young people with a mean age of 37.9 years old, a result that is similar to a study conducted with emergency nurses ^( 18)^ . 

As for the working time in the services, the participants’ mean was 6.7 years, with five years as the minimum working time among Emergency Center nurses ^( 18)^ . A previous research study found higher competence levels for nurses with more than six years of work experience ^( 19)^ . Although the literature confirms that professional competence in Nursing is improved as experience time increases ^( 20)^ , the statistical results of this study showed no association between working time and classification in the scores of the competence factors. 

The quantitative results of this study evidenced the participants’ self-assessment as “Very much competent” according to the instrument’s score, in the self-assessment of six of the seven competence factors. As length of service increases, nurses tend to evaluate their professional competence as better ^( 21)^ . This can explain the fact of the positive self-assessment evidenced in this study. 

Among the self-assessed competence factors, Factor 1 - Professional practice, as well as Factor 2 - Relationships at work, 3 - Positive challenge, 4 - Targeted action, 5 - Constructive behavior and 7 - Adaptation to change, focused on level of competence 4 - Very much competent, suggesting that basic Nursing competencies are appreciated. Corroborating these findings, an analysis carried out at emergency units in Kenya showed a similar result, where most of the nurses (84.5%) rated themselves as highly competent in the basic competencies ^( 22)^ . 

Evidence-Based Practice (EBP) grounds decision-making in care, based on the results of scientific studies and skills development, respecting the patients’ preferences and individuality. It even subsidizes the strategies that assist in qualification of the care performance and in the development of nurses’ basic competencies ^( 23)^ . 

Regarding the self-assessment of Factor 6 - Professional excellence, the quantitative analysis revealed a lower level of competence (p=0.036) in secondary care nurses when compared to their tertiary care counterparts in the self-assessment of the other factors. Similarly to the results, a Slovenian study identified the self-assessment of emergency nurses with high levels of competence and differences in them according to the care level ^( 21)^ . A high level of professional competence was also identified in Sweden, with greater variation in pre-hospital emergency care nurses, for providing self-care advice up to the contraindication of patient transportation, when compared to intensive care nurses ^( 1)^ . It is therefore perceived that the health care context can interfere with professional development ^( 1)^ . 

The Professional excellence factor involves qualified performance generating added value to the assistance provided and in professional recognition with prominence among the others ^( 14)^ . Thus, in order to maintain certain constancy in the development of competencies, it is necessary for nurses to engage with lifelong learning, so that they can refine their skills in order to achieve excellence and professional recognition ^( 24)^ . 

In an integrative review, the importance of nurses’ continuous professional development to work in emergency services was reinforced. This review pointed out that lack of training and organizational support can lead to adverse results both for the team and for the patients, such as increased adverse events, professional exhaustion and demotivation ^( 25)^ . 

Regarding Factor 2 - Relationships at work, the nurses rated themselves as very much competent in all items, evidencing positive performance in interpersonal relationships at work. A similar study identified centrality of teamwork (51%) as a motivator of a health team in the Emergency Department, emphasizing the importance of building relationships at work ^( 26)^ . 

Defined as the ability to initiate and maintain relationships, relational competence is paramount in health care ^( 27)^ . In this context, the importance of integration between interprofessional teams is emphasized for the development of collaborative and common competencies of each profession, focusing on good relations ^( 28)^ . 

The qualitative data of the “Professional recognition of nurses by the labor market” category comprised recognition by the labor market, through continuous search for knowledge associated with professional experience and ability to develop interpersonal relationships. A similar perception is found in the international literature, where competence is based on knowledge and experience at the same time ^( 29)^ . A study conducted with Nursing students corroborates these findings by asserting that it is through professional recognition and appreciation that it is possible to guarantee a harmonious environment and good interpersonal relationships ^( 27)^ . 

On the other hand, the “Deficient Permanent Education scenario in Urgency and Emergency services” category portrayed the absence of an education culture, with scarcity of practical and theoretical training opportunities, which are acquired individually and according to the work needs.

A high need for continuing education is also found internationally and strategies such as Basic Life Support (BLS), Advanced Cardiac Life Support (ACLS), Pediatric Advanced Life Support (PALS) and Emergency Screening have been instrumental in helping emergency nurses update with evidence-based practice, which is achieved by ongoing professional development ^( 22)^ . However, these strategies still seem to be insufficient and can represent an important limiting factor in access to knowledge. In southern Brazil, the SAMU professionals used their own financial resources to attend specific courses in the area ^( 30)^ . 

The integration between the quantitative and qualitative results presented a difference in Factor 6 - Professional excellence, which in the second phase of the research evidenced item “33. Uses other opportunities for their professional development” associated with the “Professional recognition of nurses by the labor market” category and items “3. Constantly updates knowledge in emergencies” and “4. Participates in realistic simulation in emergencies periodically” were associated with the “Deficient Permanent Education scenario of Urgency and Emergency services” category. In contrast, there were convergences of Factor 2 - Relationships at work with the participants’ testimonies, as shown in Figures 1 and 2, respectively. 

As they provide immediate and high-complexity assistance, urgency and emergency nurses face several challenges in order to remain updated and competent ^( 24)^ . A scoping review disclosed that the absence of structural resources, access to literature, technology, materials, lack of time to participate in educational activities or pursue research and funding or economic compensation difficulties can interfere with continuous professional development; in addition to lack of demand at work and the professionals’ lack of interest itself ^( 6, 31)^ . 

The participants’ testimonies showed that the lower level of competence of secondary care nurses was due to the deficient permanent education scenario, as well as to the lack of training opportunities using realistic simulation. In view of the absence of permanent education, most of the professionals updated themselves through private online courses, reading articles and searching graduate courses. A similar result was found among SAMU professionals ^( 30)^ . Short courses may not be sufficient to meet all the educational needs ^( 22)^ , mainly considering the need for practical training required by the urgency and emergency care scenario. 

Item “31. Participates in realistic simulation in emergencies periodically”, presented in Table 1, displayed a lower level of competence 3 - Competent, evidencing non-applicability of this strategy by the participants. Recognized as an innovative educational methodology, realistic simulation enables the direct participation of the professionals involved, being capable of correlating theory to practice ^( 32)^ . In this way, it can improve the Nursing performance levels and the critical thinking competence ^( 33)^ . Employed in the urgency and emergency scenario, it was considered useful and effective for assessing performance and skills, also providing practical learning and reflections on behaviors and teamwork ^( 34)^ . 

As a strategy for solving the critical scenario of deficient permanent education, the implementation of a specialized team to provide education stood out among the participants, as well as the financial investment for the purchase of equipment for practical training and hiring of collaborators to carry out the activities of the Center for Education in Urgencies (NEU) in the urgency and emergency context.

Originated from the National Policy for Emergency Care ( *Política Nacional de Atenção* à *s Urgências*, PNAU), the NEU aims at meeting the needs of the Unified Health System (SUS) by promoting permanent education in health for professionals and encouraging curricular adequacy in training institution ^( 30)^ . This center aims at providing training to teams at all care levels, in addition to health education for the population. 

The quantitative score in Factor 2 - Relationships at work, reached a higher level of competence (4 - Very much competent) in all items and, when contrasted with the testimonies of the secondary care participants, it confirmed the quantitative data through the “Professional recognition of nurses by the labor market” category, which emphasized the ability to develop interpersonal relationships. Optimizing interpersonal relationships favors teamwork, resulting in a beneficial and favorable professional environment for better quality care, in addition to providing cooperation on the part of the team and reducing stress at work ^( 35)^ . 

Human interaction in the health field constitutes the modern view of quality in care and emphasizes that relationships are supported by kindness, compassion, understanding and solidarity at an emotional level, in addition to attitudes of respect for the patients’ dignity, promoting a positive impact on adherence to treatments and in health results ^( 29)^ . 

Interpersonal relationships are considered a work tool and their absence can certainly and directly affect care ^( 36)^ . In this sense, a collaborative practice and interprofessional education have proved to improve the patients’ results by reducing the hospitalization times and the clinical error rates, in addition to being able to achieve the five-fold objective: better patient care, better population health, better value, better work experience and better equality in health ^( 37)^ . 

Relational competence refers to the ability to manage conflicts through a harmonious and stable relationship, which translates intellectual and emotional intelligence into interpersonal relationships, both with the context and life and in the relationship with oneself ^( 38)^ . 

Thus, the high level of competence identified may suggest presence of relational competence among the participants.

As research limitations, the convenience sample is pointed out, which does not guarantee representativeness, in addition to the size of the quantitative sample: 39 participants. The importance of sampling is highlighted because it is connected to the methodological credibility of a research study ^( 39)^ . From another perspective, international studies were observed in the scope of Nursing competencies that obtained quantitative samples between 24 and 60 participants ^( 40- 43)^ . In these cases, as well as in this study, the qualitative component collaborated by refining the initial quantitative results and enriching understanding. 

It is also important to consider that the study was carried out in a pandemic context, which made data collection extremely difficult, due to restrictions on health services and impaired the number of participants reached due to layoff of professionals, which may have reflected in the analysis of the results for the quantitative data. Therefore, it is suggested to apply the instrument again in a post-pandemic context in order to deepen research studies on the theme.

Despite this, the results obtained in this research can contribute to structuring curricula in high-complexity Nursing care, being capable of diagnosing and supporting the development of the professional Nursing practice. The findings also highlight the importance of permanent education in the context of urgency and emergency services, as investment in nurses’ training contributes to the pursuit of excellence in health care.

## Conclusion

The results of this study allowed identifying the professional competencies of urgency and emergency nurses, also enunciating weaknesses to be identified and worked on to achieve good quality care.

It was evident that the nurses considered themselves to be very much competent in terms of professional practice, relationships at work, positive challenge, targeted action, constructive behavior and adaptation to change. With regard to professional excellence, a decline in the self-assessments was observed, which showed the need for greater investments in skills and practical training opportunities for secondary health care participants.

In addition to that, in the participants’ perception, knowledge, professional experience and the ability to relate are crucial for professional success.

Therefore, the data identified represent a major contribution to the professional competences of nurses who work in emergencies, supporting the principle that, for their progress, it becomes necessary to consolidate an education and continuous professional development culture.
